# Use of Dendritic Cell Receptors as Targets for Enhancing Anti-Cancer Immune Responses

**DOI:** 10.3390/cancers11030418

**Published:** 2019-03-24

**Authors:** Md Kamal Hossain, Katherine A. Wall

**Affiliations:** Department of Medicinal and Biological Chemistry, University of Toledo, Toledo, 43614 OH, USA; mdkamal.hossain@rockets.utoledo.edu

**Keywords:** dendritic cells, Fc receptor, C-type lectin receptor, major histocompatibility complex (MHC), immunotherapy

## Abstract

A successful anti-cancer vaccine construct depends on its ability to induce humoral and cellular immunity against a specific antigen. Targeting receptors of dendritic cells to promote the loading of cancer antigen through an antibody-mediated antigen uptake mechanism is a promising strategy in cancer immunotherapy. Researchers have been targeting different dendritic cell receptors such as Fc receptors (FcR), various C-type lectin-like receptors such as dendritic and thymic epithelial cell-205 (DEC-205), dendritic cell-specific intercellular adhesion molecule-3-grabbing non-integrin (DC-SIGN), and Dectin-1 to enhance the uptake process and subsequent presentation of antigen to T cells through major histocompatibility complex (MHC) molecules. In this review, we compare different subtypes of dendritic cells, current knowledge on some important receptors of dendritic cells, and recent articles on targeting those receptors for anti-cancer immune responses in mouse models.

## 1. Introduction

Checkpoint inhibitors (CTLA-4, PD-1/PD-L1), adoptive cell transfer, monoclonal antibodies and cancer vaccines are among the most popular cancer immunotherapy modalities available so far. Some of these therapies are either used alone or as adjuvant therapy in combination with other conventional therapy. Dendritic cell (DC)-based vaccines drew attention because of these cells’ special abilities in coordinating both innate and adaptive immunity and in inducing tumor specific effector cells and immune memory cells. DCs, specialized antigen presenting cells (APCs), are known as sentinels of the immune system and play a central role in initiating or regulating immune responses. DCs were discovered by Ralph Steinman in 1973 and constitute about 1% of mononuclear cell compartments [1]. They are about 100-fold more potent in initiating adaptive immune responses compared to macrophages and monocytes [2]. DCs are found in almost all tissues, mostly in skin, lung, stomach and intestine [3]. They reside in peripheral tissue in an immature stage and are specialized in recognizing invading pathogens or antigens. However, they are weak stimulators at this stage due to their low levels of major histocompatibility complex (MHC) molecules, adhesion molecules and co-stimulatory molecules. In the absence of infection or inflammation, when the antigens are mostly self-antigen, DCs are tolerogenic. When infected, due to the presence of danger signals, immature DCs undergo a maturation phase in the presence of co-stimulation [4]. During the maturation phase, DCs upregulate different chemokine receptors (CCR7 and CD62L) that lead the DCs to migrate to the secondary lymphoid tissue and induce immune responses by interacting with B and T cells.

Exogenous antigens are usually captured and processed by DCs and presented through their MHC II molecules present on the cell surface. MHC II molecules have two chains (α and β) in the endoplasmic reticulum (ER) that are stabilized by invariant chain (Ii). The complex of MHC II and an invariant chain is transported through the Golgi to a compartment called the MHC II compartment. At low pH, cathepsin S and cathepsin L digest the invariant chain and produce class II associated Ii peptide (CLIP). Endosomally digested peptides replace CLIP and MHC II is then ready to present these peptides on the cell surface to CD4^+^ T cells [5,6].

Endogenous antigens are cleaved by the proteasome into peptides, transported by the transporter associated with antigen presentation (TAP), and eventually are presented on MHC I molecules. MHC I molecules are generally expressed by nucleated cells and contain two types of chains: heavy chain and β2-microglobulin. Once the antigen is degraded by the proteasome, the peptides go to the ER via the TAP [7,8]. Before binding to peptide, MHC I molecules are stabilized by chaperone proteins, and when peptides bind to MHC I molecules, those chaperone proteins are released [9]. The peptide-MHC I complexes then leave the ER and present peptides on the cell surface to the CD8^+^ T cells [10].

Immature dendritic cells, after encountering antigen, are stimulated and activated to become mature DCs. Mature DCs are also equipped with co-stimulatory molecules (CD80, CD86 and CD40), that provide a second signal necessary for T cell activation. DCs then travel to the secondary lymphoid organs from peripheral tissue to interact with T cells. CD4^+^ T cells are activated to become Th cells and release several cytokines to direct the immune response, e.g., interleukin-12 (IL-12) promotes a Th1 immune response that is directed towards cellular immunity and IL-4 promotes a Th2 immune response that induces a humoral immune response. Th1 cells primarily produce interferon-γ (IFN-γ) and IL-2 that is responsible for activation of T cells and inflammation. On the other hand, Th2 cells promote the secretion of IL-4 and IL-10 that are responsible for B cell activation to produce antibodies [11,12]. CD8^+^ T cells are activated to release tumor necrosis factor (TNF) and to become cytotoxic T cells (CTL) that kill tumor cells [13].

**Figure 1:**
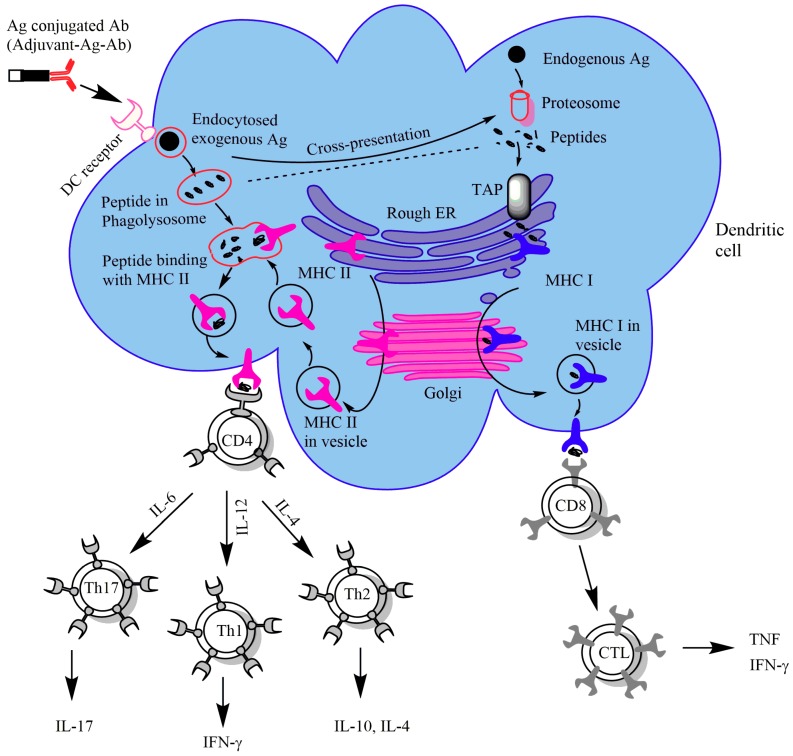
Antigen processing and presentation scheme through an antibody-based dendritic cell (DC) targeting vaccination approach. Exogenous antigens are degraded and processed for presentation on DC surface-associated major histocompatibility complex (MHC) II molecules to CD4^+^ T cells. Endogenous antigens are degraded and processed for presentation on MHC I molecules to CD8^+^ T cells.

Antibody-based DC targeted vaccination is a promising approach in vaccine development [14]. Antigen is bound with antibody and targeted to a dendritic cell receptor for internalization, processing and presentation (Figure 1). Antigen can be delivered through several receptors of dendritic cells including C-type lectin receptors (CLRs) (mannose receptor, DEC-205, Dectin-1, DNGR-1 and others), Fc receptors and others. Choosing which receptor to be targeted is a great challenge for an antibody-based DC targeting approach since there is little consensus over which receptor elicits more MHC I or MHC II antigen presentation. However, it was found that the amount of antigen, the speed of internalization, and receptor expression level did not impact MHC I or MHC II antigen presentation efficiency [15].

A successful therapeutic vaccination approach relies heavily on antigen loading and priming of T cells through activation of dendritic cells. This review highlights the importance of different kinds of dendritic cell receptors crucial for an effective antibody-targeted vaccination approach and the recent findings associated with targeting those receptors. We discuss how these findings may have an impact on our understanding of the receptor binding interaction focusing on DCs. We begin, however, by reviewing different dendritic cell subsets and their biology, receptors present on different subsets and targeting those receptors through corresponding antibody–antigen conjugation approaches.

## 2. Dendritic Cell Subsets and Receptors

**Figure 2:**
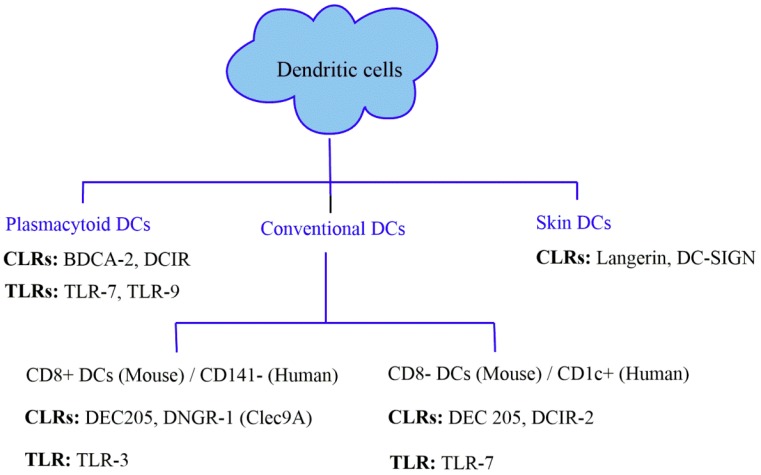
Different DCs subsets with corresponding differentially-expressed receptors.

Vaccination heavily relies on inducing B cell and antibody responses. However, inducing effective T cell immune responses remains somewhat challenging. Hence, targeting antigens to different dendritic cell receptors is essential for generating cellular immunity via improving targeting strategies. Dendritic cells can be divided into two major subsets: plasmacytoid dendritic cells (pDCs) and conventional or myeloid dendritic cells (mDCs). Different subsets have different receptors that could be targeted for antigen presentation and efficient vaccine preparation (Figure 2).

Plasmacytoid dendritic cells (pDCs): pDCs usually circulate in the blood and are prevalent in spleen, thymus, bone marrow, and in certain peripheral tissues [16]. FLT-3 ligand (FLT-3L) is the differentiation factor that controls the proliferation of pDCs from hematopoietic progenitor cells [17]. It is believed that the main function of pDCs is in inducing innate immunity [18]. They utilize Toll-like receptors TLR receptors such as TLR7 and TLR9 in fighting viral infection to produce large amounts of type I interferon (IFNs) [19]. pDCs are generally known for having poor antigen presentation capacity even though they produce MHC II molecules continuously [20]. However, pDCs were also found to have enhanced antigen uptake and presentation from apoptotic cells, immune complexes and viruses [21]. pDCs have also been found to be associated with immune tolerance [22]. They are able to take up exogenous antigens through the Fcγ receptors (FcγR)II receptors and present them to CD4^+^ T cells [23]. Besides expressing TLRs and FcγRII, pDCs also express CLRs such as blood dendritic cell antigen-2 (BDCA-2) and dendritic cell immunoreceptor (DCIR). Targeted delivery of antigen to the DCIR receptor results in both antigen presentation and IFN-α production [17,24].

Conventional dendritic cells (cDCs): cDCs are found in peripheral and lymphoid tissue, blood and other tissues. They are subdivided into either migratory or stationary dendritic cells. Migratory DCs capture antigen and migrate from peripheral tissues such as skin, liver, kidney, lung and intestinal tract to lymphoid organs and interact with T cells. On the other hand, stationary DCs lack the ability to move and stay in the lymphoid organ such as lymph node, spleen and thymus [25]. Mouse cDCs consist of two major subsets: CD8^+^ DCs and CD8^-^ DCs. Their human counterparts are known as CD141^+^ (BDCA3^+^) and CD1c^+^ (BDCA1^+^). CD8^+^ DCs show high expression of CLRs such as DEC-205, which is responsible for antigen capture and cross-presentation on MHC I molecules [26,27]. However, DEC-205 is also expressed on Langerhans cells, B cells and dermal DCs but in low amount. CD8^+^ DCs also have a high expression level of DNGR 1 (Human Clec9A), another C-type lectin receptor which has the potential of facilitating the process of antigen presentation [28]. CD8^-^ DCs do not express DNGR 1 but have a low level of DEC205 [29]. They express another CLR, DCIR2 that is specialized for antigen capture and presentation on MHC II molecules [30]. There are also differences between these two subsets in pattern recognition receptors (PRR) while responding to invading pathogen. Toll like receptor-3 (TLR-3) is highly expressed on CD8^+^ DCs but is absent on CD8^-^ DCs [31]. It recognizes dsRNA of the viral pathogen and activates transcription factor interferon regulatory factor-3 (IRF-3) and NF-κB [32]. In contrast, TLR-7 is absent on CD8^+^ DCs but present on CD8^-^ DCs. It recognizes single-stranded RNA of the viral genome such as HIV [33,34].

Skin dendritic cells: Skin also contains some important dendritic cell subsets such as Langerhans cells (LCs). LCs are found in the epidermis of both mice and humans and are responsible for the capture and transportation of antigen from the periphery to the lymph node for activation of memory and naïve T cells. LCs can be recognized by the expression of langerin, a C-type lectin receptor found on skin DCs [35]. Another skin DC subset is called dermal DCs, which express another CLR, dendritic cell-specific intercellular adhesion molecule-3-grabbing non-integrin (DC-SIGN), found mainly in dermis [35].

## 3. Targeting Different DCs Receptors for Vaccine Development

C-type lectin receptors (CLRs) are some of the other important DC receptors capable of recognizing sugar moieties from pathogens. Making glycosylated vaccines and targeting towards these receptors has been very difficult due to the presence of too many CLRs recognizing a sugar. Hence, antibodies have mainly been utilized to specifically target a certain receptor to induce strong humoral and cellular immune responses. Another DC receptor, Fcγ receptor (FcγR), is also very efficient for targeted delivery of antigen by antibodies.

### 3.1. C-type Lectin Receptors

CLRs, found in either transmembrane or soluble form, are generally characterized by the presence of carbohydrate recognition domains (CRD). Unlike toll-like receptors (TLRs), which recognize pathogen associated molecular patterns (PAMPs) such as lipopolysaccharide, CLRs recognize glycan structures on the pathogen through their CRDs [36]. It was initially assumed that calcium ion is required for binding with carbohydrate and thus the term C-type lectin receptors. The CLR family includes lymphocyte lectins, collectins, selectins and 14 other groups.

**Figure 3:**
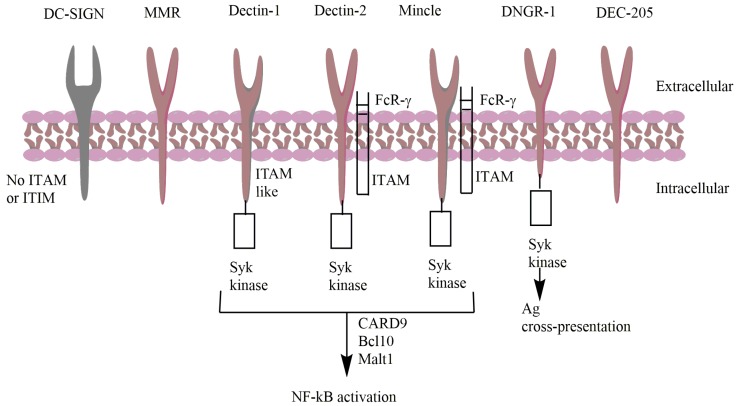
Signaling pathway of different DCs receptors (CLRs). Downstream signaling leads to the activation of NF-κB and pro-inflammatory cytokines.

Antigen presenting cells (APCs) such as macrophages and dendritic cells express CLRs that serve as pattern recognition receptor (PRRs) and bind to PAMPs or self-antigen released from dead cells [37]. Sometimes CLRs lack the need for calcium ion but are able to recognize carbohydrate and some other ligands such as proteins and lipids. These are commonly referred to as C-type lectin-like receptors (CTLRs). e.g., Dectin 1 and 2 [38]. Some CLRs serve as phagocytic receptors only, whereas other CLRs activate a signaling cascade to initiate immune responses. CLR engagement leads to the activation of a tyrosine kinase such as Syk via immune-receptor tyrosine-based activation motifs (ITAMs) and subsequently, through downstream signaling, activates the NF-κB pathway and initiates cellular immune responses (Figure 3) [39]. In contrast, CLRs such as DC immunoreceptor (DCIR) and myeloid c-type lectin-like receptor contain immune-receptor tyrosine based inhibitory motifs (ITIMs), which inhibit cellular activation to prevent uncontrolled immune responses [38].

Antibody-mediated CLR targeting has been a very successful approach for many years. The most commonly used CLRs for antibody-targeted approaches are the mannose receptor, 205 kD membrane protein (DEC-205), Dectin-1 and 2, dendritic cell natural killer lectin group receptor-1 (DNGR-1), and DC-specific ICAM-3 grabbing non-integrin (DC-SIGN) (Figure 2).

#### 3.1.1. Mannose Receptor (MR)

The mannose receptor (MR), also known as CD206, is a CLR mostly present on macrophages and immature dendritic cells [40]. This receptor contains eight carbohydrate recognition domains (CRD), a fibronectin type II domain, and a cysteine-rich N-terminal domain on its extracellular region [41]. They are capable of recognizing mannose, fucose and N-acetylglucosamine residues present on the surface of microorganisms [42]. They also play a role in antigen uptake and presentation through binding of glycolipid antigens from lipoarabinomannan (LAM) to MR receptors on immature DCs. This enables antigens to be internalized and transported to endocytic vesicles for presentation to T cells in complex with CD1b [43].

Mature dendritic cells, however, take the processed antigen to effector cells in lymphoid organs via the cysteine-rich domain and thus induce adaptive immune responses [43]. Immunizing mice with the human tumor antigen MUC1 fused with oxidized mannan led to the induction of tumor-specific CD8^+^ T cells and elimination of MUC1^+^ tumor cells. In contrast, immunization with reduced mannan-MUC1 fusion protein led to poor protection and a weak immune response [44]. It was suggested that uptake of antigen and DC maturation could vary depending on which DC receptors interacted with the antigen. MUC1 fusion protein conjugated with oxidized mannan has been in a Phase III clinical trial and showed significant reduction of recurrence rate in breast cancer patients compared to the placebo group [45]. In a different study, it was found that two TLR agonists (CpG and Poly-ICLC) were necessary to induce a Th1 protective immune response. Human chorionic gonadotropin beta chain (hCGβ) was used as an antigen and fused with anti-MR antibody B11 [46]. Co-administration of those adjuvants with the fused B11-hCGβ enhanced the accumulation of B11-hCGβ loaded DCs in the lymph node T cell areas.

#### 3.1.2. DEC-205

DEC 205 is a type I endocytic receptor protein mostly present on thymic medullary DCs, cortical thymic epithelium, or peripheral DCs in mouse [47]. In humans, DEC205 is highly expressed on myeloid blood DCs and monocytes and at low levels on T cells, NK cells and plasmacytoid blood DCs [48]. DEC205 belongs to the macrophage mannose receptor family of CLR type receptor and is characterized by its cysteine-rich N-terminal domain, fibronectin type II domain and ten carbohydrate recognition domains [49].

Even though DEC205 is present on other types of cells, DEC205 present on DCs has been targeted with corresponding anti-DEC205 monoclonal antibody conjugated with antigen for T cell activation. Many antigens so far have been delivered to DEC205 DCs in order to induce humoral and cellular immune responses. Among them, most notable are ovalbumin (Ova), HER2/neu, HIV gag, survivin and others [50,51,52,53]. For example, it has been reported that targeting CD8^+^ DCs with Ova-conjugated anti-DEC205 antibodies resulted in robust MHC- I cross-presentation to CD8^+^ T cells. Also, targeting CD8^-^ DCs with this conjugate elicits high antigen (Ova) presentation to MHC class II molecules [50,54]. However, targeting DCs through DEC205 without adjuvant led to the induction of tolerance, whereas in the presence of adjuvant, it led to the generation of an antigen-specific cellular immune response [52,55,56].

When CpG oligodeoxynucleotide (CpG-ODN); a synthetic TLR9 ligand, was used as an adjuvant targeted to the receptor DEC205, it enhanced antigen uptake, whereas mice deficient in DEC205 had impaired DC maturation and cytokine production [57]. In the presence of maturation stimuli, targeting HIV Gag p24 to DEC205 using anti-DEC205 fusion mAb induced gag-specific T helper 1 (Th1) and CD8^+^ T cells in a murine model [52].

A fusion protein, such as using a DEC205 specific antibody to fuse with antigen, can be an excellent option for targeting towards DCs as they have shown increased efficiency of antigen delivery and DC maturation [58]. A DNA vaccine encoding the Hc domain of *Botulinum* neurotoxin serotype A (AHc) fused with a single-chain fragment variable (scFv) antibody fragment against DEC205 was found to generate stronger humoral and lymphocytic proliferative responses with DC maturation [59]. Using scFv offers some advantages over the whole antibody. Due to the smaller size, scFv enter into the tissue much more easily than whole antibody [60]. Since they lack an Fc domain, they do not bind to Fc receptors and hence provide DEC205 specific antigen delivery by reducing nonspecific uptake of antigen [61].

Targeting *T. gondii* surface antigen SAG1 to DCs using an scFv antibody fragment against DEC205 led to improved local and systemic humoral and cellular immune responses [62]. Selective targeting of ovalbumin (Ova) antigen to DCs using recombinant scDEC-Ova also resulted in much higher antigen uptake and presentation to both CD8^+^ and CD4^+^ T cells compared to soluble Ova. The same group also found strong and long-lasting specific CD4^+^ T cells when they targeted DCs with scDEC-Gag protein plus poly ICLC vaccine [63]. In a recent study, targeting DEC205 with a DC specific adenoviral vector expressing human glioma specific antigen showed prolonged survival in a murine glioma tumor model [64]. However, most of the adjuvants used in the murine model were not suitable for human use. A non-toxic cholera B subunit (CTB) was used as an adjuvant successfully with anti-DEC205-Ova as a DC targeted vaccination. This approach promoted local and systemic protection with CD4^+^ T cell expansion and priming of Th1 and Th17 cells [65].

#### 3.1.3. Dectin-1 and 2

Dectin-1 is a transmembrane receptor expressed mainly by myeloid cells in both mice and humans. It is found mostly on monocytes, macrophages, DCs and some lymphocyte cells. It has an extracellular C-type lectin-like domain (CTLD) and an intracellular immunoreceptor tyrosine-based activation (ITAM) like motif [66,67]. Dectin-1 recognizes β-glucans that are found on fungi and on some bacteria [68,69]. Upon binding with its ligand, Dectin-1 initiates phagocytosis and activates Src and Syk kinase through its ITAM motif. Through downstream signaling, NF-κB becomes activated and the cell secretes cytokines [70].

An interaction of Dectin-1 with β-glucan is crucial for inducing cellular immune responses including DC maturation, antigen uptake and phagocytosis, and production of cytokines and chemokines such as TNF, CXCL2, IL-6 and IL-10 [67]. It is reported that Dectin-1 ligation with fungal β-glucan induces the differentiation of Th-1 and Th-17 CD4^+^ T cells [71,72]. Dendritic cells activated by the Dectin-1 agonist Curdlan are very efficient in promoting expansion and differentiation of cytotoxic T lymphocytes (CTL) that protect mice from tumor challenge. This agonist is also found to be an adjuvant for CTL cross-priming and helps in stimulating CD8^+^ T cell responses [73].

Dectin-2, another type-2 transmembrane receptor, recognizes α-mannan, a carbohydrate that is found on fungi. Upon binding, spleen tyrosine kinase is recruited to ITAM and through downstream signaling leads to the production of inflammatory cytokines [74]. Besides being anti-inflammatory, Dectin-2 is also reported to have a role in suppressing liver metastasis through CD11b F4/80 Kupffer cells [75,76].

#### 3.1.4. DC-SIGN

DC-specific ICAM-3 grabbing non-integrin (DC-SIGN) is a type 2, mannose-specific C-type lectin that induces specific immune responses upon recognition of glycans through its CRD. It consists of an extracellular CTLD, a single transmembrane domain, and an N-terminal cytoplasmic tail [77]. DC-SIGN recognizes a variety of fucosylated glycans and mannose structures and hence is able to bind different pathogen-derived and some self-glycoproteins [78]. It is generally present on immature dendritic cells in peripheral tissue, and on mature DCs in lymphoid organs and is absent on follicular DCs [79]. DC-SIGN mediates binding of DCs to ICAM-3 on T-lymphocytes for the activation of T cells [80].

For DC targeting, DC-SIGN-specific antibodies are either coupled with antigen or conjugated in a multivalent fashion with the surface of nanoparticles [81,82]. Targeting Ova antigen coupled with anti-DC-SIGN antibodies to DC-SIGN elicited Ova-specific strong and persistent CD4^+^ and CD8^+^ T cell responses that protected from Ova expressing *Listeria* [83]. Administration of anti-DC-SIGN antibodies conjugated with Tetanus Toxoid (TT) in a humanized mouse model also elicited an antigen-specific stimulatory T cell response without the need of excess adjuvant [84]. In another study, antibody conjugated antigen targeted to DC-SIGN was compared with coupling antigen to a cell penetrating peptide. Both approaches were found to be almost equally efficient at causing cross-presentation of antigens [85].

#### 3.1.5. DNGR-1

Dendritic cell natural killer lectin group receptor-1 (DNGR-1) or Clec9A, a type II transmembrane CLR, acts as a damage-associated molecular pattern receptor. When cell death or tissue injury occurs, damage-associated molecular patterns (DAMPs) such as proteins, nucleic acids or metabolites are released. In the process of removing DAMPs, the antigens are targeted to special dendritic cells such as conventional dendritic cells (cDCs), the most efficient cross-presenting DCs to target CD8^+^ T cells [86]. DNGR-1 contains a single extracellular CTLD and a cytoplasmic tail with a hem-ITAM motif that allows binding to spleen tyrosine kinase (Syk) (Figure 3). DNGR-1 detects the filamentous form of actin (F-actin) exposed on apoptotic cells when the cell membrane is ruptured [87]. DNGR-1 is highly expressed on cross-presenting CD8^+^ DCs (in human, CD141^+^ DCs) and has low expression levels on plasmacytoid DCs (in mice) [28].

When anti-DNGR-1 antibody was covalently coupled with Ova protein and targeted to DCs in-vivo via DNGR-1 along with an adjuvant, it induced a potent CTL response that could inhibit Ova-expressing lung metastasis [88]. In another study, an anti-DNGR-1 antibody coupled with tumor-associated antigen MUC1 was injected into human MUC1-expressing transgenic mice, a MUC1 specific immune response was found that could significantly delay the growth of MUC1-expressing tumors [89]. It was reported that in the absence of adjuvant, antigen targeting to DNGR-1 produces a weak antibody response and does not lead to primed CD4^+^ T cells but, rather leads to the conversion of naïve CD4^+^ T cells to Foxp3^+^ regulatory T cells [90]. However, a strong humoral immune response was also found even without adjuvant when targeting DNGR-1 through an antibody-dependent antigen targeting approach [28,91].

Myosin II, an F-actin associated motor protein has been recently found to synergize with the binding of F-actin to DNGR-1 [92]. Antigen particles bearing F-actin and myosin II were efficiently taken up by DNGR^+^cDCs and cross presented to CD8^+^ T cells, whereas, myosin II-deficient necrotic cells could not stimulate DNGR-1.

### 3.2. Fc Receptors

Exploiting Fc receptors for interaction with antibody Fc domains for uptake and processing of antigen by dendritic cells has been a successful approach. There are different Fc receptors for different immunoglobulins present, such as the Fcα receptor (FcαR) for IgA, Fcγ receptor (FcγR) for IgG, and Fcε receptor (FcεR) for IgE. Among them, FcγR is the receptor very well known for its capacity to function as a regulator of the immune response and responsible for the immune-complex mediated maturation of DCs [93]. IgG molecules bind to the desired antigen via two fragment antigen binding sites (Fab) through complementary determining regions (CDR) and the Fc domain makes a crosslink with the specific receptors (Fcγ receptors) of APCs. This leads to the activation of effector functions including phagocytosis, ADCC, cytokine and chemokine production, and modulation of T and B cells [94].

**Figure 4:**
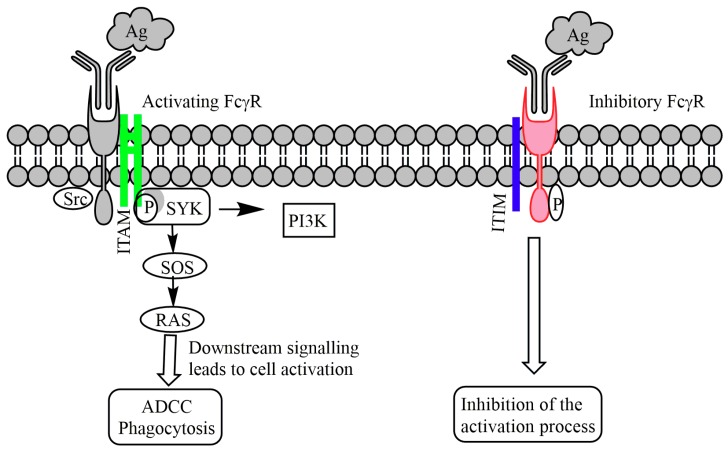
Signal transduction by Fcγ receptors (FcγR) immune-receptor tyrosine-based activation motifs (ITAM) or inhibitory motifs (ITIM). Activating FcγR crosslinking by IgG bound to antigen leads to the phosphorylation of ITAM by Src and SYK kinases. This allows SYK kinase to activate other pathways such as RAS and PI3K. This, in turn, increases the cellular calcium level and phagocytosis of the immune complex. Inhibitory FcγR (FcγRIIB) contains an ITIM and inhibits the activation process.

FcγRs are subdivided into type I and II based on their structural binding stoichiometry and Fc domain binding sites [95,96]. Type I is also divided into activating or inhibitory based on their signaling motif (Figure 4). FcγRI, FcγRIIa, FcγRIIc and FcγRIIIa are among activating receptors due to the presence of ITAM motifs whereas, FcγRIIb is the only inhibitory receptor containing an ITIM motif in its intracellular region [97]. Binding of Fc with an activating receptor triggers the phosphorylation of ITAM, and through downstream signaling activates Src and Syk family kinases and pro-inflammatory signaling pathways [96,97]. On the other hand, binding of Fc with an inhibitory receptor triggers the phosphorylation of ITIM, and through downstream signaling inhibits the activation of Src kinases and phospholipase C γ (PLCγ). This inhibits the production of pro-inflammatory cytokines. Type II FcγR includes DC-SIGN and CD23, which also belongs to the C-type lectin receptor family [95]. They have the capacity to interact with other ligands such as a carbohydrate (e.g., mannose for DC-SIGN receptor) along with regular Fc domains.

To understand the Fc-FcγR interaction, the expression pattern of FcγR and the different binding profiles of isotypes of IgG need to be understood. There are some differences in the expression pattern of activating FcγR receptors present on mouse and human cells [93]. FcγRI, present in both humans and mice, is expressed on monocytes, macrophages and DCs in humans, whereas, in mice it is present mainly on monocytic DCs and some subsets of monocytes and macrophages. FcγRIIa is expressed mainly on human cells and is present on myeloid cells including granulocytes, monocytes macrophages and DCs. FcγRIII in mice is expressed on NK cells, monocytes, macrophages, granulocytes and DCs, and FcγRIIIa in humans is expressed on NK cells, macrophages and some monocytes in the spleen. FcγRIIIb is another receptor that is not well characterized and is present on granulocytes such as neutrophils and eosinophils. However, inhibitory receptor FcγRIIb is present in both mice and humans and is expressed on B cells, DCs and some macrophages in mice. Other than those receptors, a neonatal receptor (FcRn) and intracellular tripartite motif-containing 21 (TRIM21) are also present in humans, and they are expressed on almost all cells, especially myeloid cells [98,99].

Binding affinities and specificities of IgG isotypes to various FcγR receptors are also different between mice and humans due to the differences in the amino acid sequences of different IgG subtypes [96]. In humans, IgG1 and IgG3 bind almost all FcγRs and are potent inducers of cell mediated effector function [100]. However, IgG2 only binds with FcγRIIa and has lower binding affinity for FcγRIIIa. IgG4 has nanomolar binding affinity for FcγRI and micromolar affinity for FcγRII. In mice, IgG1 bind to FcγRIIb and FcγRIII and IgG3 only bind to FcγRI. However, IgG2a and IgG2b bind to almost all FcγRs.

Fc domains link the innate immune response to the adaptive immune response through immune complexes and through stimulating T cells. Targeting a specific antigen, *Francisella tularensis* (Ft), an agent responsible for tularemia, to FcγR intranasally as an mAb-inactivated Ft immunocomplex enhanced the protection against this specific antigen (Ft) [101]. The researchers demonstrated that the enhanced immunogenicity was not due to adjuvant but due to more effective DC maturation and enhanced antigen processing and presentation. In a different report, the same lab also showed that this similar immunization technique increased the number of activated dendritic cells in the lungs of immunized mice as well as in the frequency of IFN-γ secreting memory CD4^+^ T cells [102]. It was also shown by a different group that targeting FcγR with Ova-IgG complex was ten times more efficient, in-vivo, in activating antigen specific CD8^+^ T cells than soluble Ova [103].

We and others have targeted the FcγR of dendritic cells for effective uptake and presentation of MUC1 antigen [104,105,106,107]. We have utilized natural anti-rhamnose (Anti-Rha) antibodies to form an immune complex with a Rha-containing MUC1 vaccine, in-vivo [108]. The difference from other studies discussed earlier was that instead of conjugating the antibody with antigen prior to immunization, we injected anti-Rha antibody intraperitoneally in mice one hour before immunizing with Rha-vaccine. This approach more strongly induced both humoral and cellular immune responses to MUC1.

### 3.3. Targeting Methodologies

Table 1 below lists representative recent publications found in a PubMed search in the field of DC targeting via the discussed receptors.

## 4. Conclusions

In conclusion, targeting antigen to DCs has been a very exciting approach to generate effective antibody responses and generating effective T cells for a long time. DEC205, Fcγ, DNGR-1 and Dectin-1 receptors have been considered as potential receptors for inducing both humoral and cellular immune responses. Interestingly, some of the receptors even resulted in enhanced antigen presentation and T cell activation without the need for adjuvant. However, targeting a single DC receptor for an Ab based vaccination approach may not be quite as successful in human clinical trials as in murine models since DCs may not receive enough stimulation by targeting a single specific receptor. Also, there is no consensus yet on which DC receptor would be a choice of priority in terms of targeting antigen. There are some studies which reported that targeting multiple receptors instead of a single receptor could give a synergistic boost to generate activated T cells. Using checkpoint inhibitors in addition to in-vivo targeting of DC receptors may be a successful approach in the long run to elicit enhanced immune responses against a particular antigen. Finally, more extensive understanding of DC antigen processing and presentation pathways are required to decipher the required amount of antigen loading, speed of internalization and pathway to design more effective DC targeting vaccination.

## Figures and Tables

**Table 1:** Dendritic cell targeting through different receptors in vivo. References listed are representative publications found in a PubMed search in the related field in recent years. *

Receptors	Antigens	Adjuvant	Mice	Immunization	Results	Ref.
DEC205	1. SAG-1 (*T. gondii*)	1. Poly (I:C)	1. CBA/J (H-2^k^)	1. IN & SubQ	1. Th1 (IFN-γ, IL-2, IgG2a, IgA)	[62]
	2. HIV-1 gagP24	2. Poly-ICLC	2. CXB6 F1	2. SubQ	2. CD4^+^ T cells	[109]
	3. Ova	3. CTB	3. C57BL/6 and Tg GFP	3. ID	3. CD4^+^ T cells, Th1, Th17	[65]
	4. AHc	4. No adjuvant	4. Balb/c	4. IM	4. IgG1, IgG2a, Mature DC	[59]
	5. RSV fusion protein	5. No adjuvant	5. Balb/c	5. IM	5. Th1, CD8^+^ T cells	[110]
MR	1. MUC1	1. IFA	1. Balb/c	1. IP	1. IgG1, IgG2a, IgG2b	[111]
	2. MAA	2. CpG and Poly(I:C)	2. C57Bl/6 and Tg OT-II	2. SubQ	2. IgG, IgG1, IgG2c, Th1	[112]
	3. hCGβ	3. CpG and Poly-ICLC	3. hMR-Tg and WT	3. SubQ	3. Th1	[46]
Dectin-1	1. Ova	1. Curdlan	1. C57BL/6	1. SubQ	1. IgG1, IgG2a, IgG3, IgA, CD4^+^ and CD8^+^ T cells	[113]
	2. Diphtheria toxin (CRM_197_)	2. β-glucans hexamer	2. Balb/c	2. ID	2. IgG1, IgG2a	[114]
DC-SIGN	1. Ag85B (Mtb)	1. CTA1-DD and zymosan	1. hSIGN and P25ktk	1. IP	1. CD4^+^ T cells, IFN-γ	[115]
	2. triMN-LPR	2. triMN-LPR as intrinsic adjuvant	2. C57BL/6J (H-2b) CD11c-YFP	2. ID	2. DC upregulation, CD8^+^ T cells	[116]
DNGR-1	1. Ova	1. Poly (I:C)	1. C57BL/6J WEHI, Clec9A^−/−^, IRF8^−/−^, Batf3^−/−^ mice	1. IV	1. IgG, CD4^+^ and CD8^+^ T cells	[117]
	2. Anti-Clec9A	2. With or without Poly (I:C)	2. C57BL/6	2. IV	2. Ig	[91]
	3. Ova	3. With or without anti-CD40	3. C57BL/6, B6.SJL and OT-I × *rag^−/−^*	3. IV	3. CD8^+^ T cells, IFN-γ	[88]
	4. MUC1	4. Anti-CD40 and Poly (I:C)	4. MUC1xA2K/b Tg	4. SubQ	4. CD8^+^ T cells, IFN-γ	[89]
FcγR	1. MUC1-Tn	1. Pam3CysSK4	1. C57BL/6	1. IP	1. IgG, IFN-γ, CD4^+^ and CD8^+^ T cells	[108]
	2. E75 (HER-2)	2. GM-CSF	2. FVB/N-Tg(MMTV-neu), Balb/c	2. IP	2. CTL	[118]
	3. iFT	3. No Adjuant	3. C57BL/6, B6.129S1 Il12a^tm1Jm^/J	3. IN	3. DC upregulation, IFN-γ secreting CD4^+^ T cells	[102]
	4. gp120_αgal_/p24	4. Ribi adjuvant	4. α1,3GT KO	4. IP	4. IgG, CD4^+^ and CD8^+^ T cells	[119]
	5. α-gal	5. Ribi adjuvant	5. α1,3GT KO	5. IP	5. IgG, CD4^+^ and CD8^+^ T cells	[120]

* Abbreviations: AHc = recombinant Hc of *Clostridium botulinum* neurotoxin serotype A, MAA = melanoma-associated antigens, iFT = inactivated *Francisella tularensis,* CTB = cholera toxin B subunit, IN = intranasal, IP = intraperitoneal, ID = intradermal, IM = intramuscular, IV = intravenous, SubQ = subcutaneous.

## References

[1] Ljunggren H.G. (2012). Dendritic cells, dendritic cell-based vaccines and Ralph Steinman. J. Intern. Med..

[2] Steinman R.M., Witmer M.D. (1978). Lymphoid dendritic cells are potent stimulators of the primary mixed leukocyte reaction in mice. Proc. Natl. Acad. Sci. USA.

[3] McMenamin P.G. (1999). Distribution and phenotype of dendritic cells and resident tissue macrophages in the dura mater, leptomeninges, and choroid plexus of the rat brain as demonstrated in wholemount preparations. J. Comp. Neurol..

[4] Reis e Sousa C. (2004). Activation of dendritic cells: Translating innate into adaptive immunity. Curr. Opin. Immunol..

[5] Blum J.S., Wearsch P.A., Cresswell P. (2013). Pathways of antigen processing. Annu. Rev. Immunol..

[6] Germain R.N. (1994). MHC-dependent antigen processing and peptide presentation: Providing ligands for T lymphocyte activation. Cell.

[7] Elliott T. (1997). Transporter associated with antigen processing. Adv. Immunol..

[8] Momburg F., Hammerling G.J. (1998). Generation and TAP-mediated transport of peptides for major histocompatibility complex class I molecules. Adv. Immunol..

[9] Paulsson K., Wang P. (2003). Chaperones and folding of MHC class I molecules in the endoplasmic reticulum. Biochim. Biophys. Acta.

[10] Zhang Y., Williams D.B. (2006). Assembly of MHC class I molecules within the endoplasmic reticulum. Immunol. Res..

[11] Chen P., Liu X., Sun Y., Zhou P., Wang Y., Zhang Y. (2016). Dendritic cell targeted vaccines: Recent progresses and challenges. Hum. Vaccin. Immunother..

[12] Berger A. (2000). Th1 and Th2 responses: What are they?. BMJ.

[13] Wohlleber D., Kashkar H., Gartner K., Frings M.K., Odenthal M., Hegenbarth S., Borner C., Arnold B., Hammerling G., Nieswandt B. (2012). TNF-induced target cell killing by CTL activated through cross-presentation. Cell Rep..

[14] Keler T., He L., Ramakrishna V., Champion B. (2007). Antibody-targeted vaccines. Oncogene.

[15] Reuter A., Panozza S.E., Macri C., Dumont C., Li J., Liu H., Segura E., Vega-Ramos J., Gupta N., Caminschi I. (2015). Criteria for dendritic cell receptor selection for efficient antibody-targeted vaccination. J. Immunol..

[16] Tacken P.J., Figdor C.G. (2011). Targeted antigen delivery and activation of dendritic cells in vivo: Steps towards cost effective vaccines. Semin. Immunol..

[17] McKenna K., Beignon A.S., Bhardwaj N. (2005). Plasmacytoid dendritic cells: Linking innate and adaptive immunity. J. Virol..

[18] Reizis B., Bunin A., Ghosh H.S., Lewis K.L., Sisirak V. (2011). Plasmacytoid dendritic cells: Recent progress and open questions. Annu. Rev. Immunol..

[19] Swiecki M., Colonna M. (2015). The multifaceted biology of plasmacytoid dendritic cells. Nat. Rev. Immunol..

[20] Young L.J., Wilson N.S., Schnorrer P., Proietto A., ten Broeke T., Matsuki Y., Mount A.M., Belz G.T., O’Keeffe M., Ohmura-Hoshino M. (2008). Differential MHC class II synthesis and ubiquitination confers distinct antigen-presenting properties on conventional and plasmacytoid dendritic cells. Nat. Immunol..

[21] Hoeffel G., Ripoche A.C., Matheoud D., Nascimbeni M., Escriou N., Lebon P., Heshmati F., Guillet J.G., Gannage M., Caillat-Zucman S. (2007). Antigen crosspresentation by human plasmacytoid dendritic cells. Immunity.

[22] Ochando J.C., Homma C., Yang Y., Hidalgo A., Garin A., Tacke F., Angeli V., Li Y., Boros P., Ding Y. (2006). Alloantigen-presenting plasmacytoid dendritic cells mediate tolerance to vascularized grafts. Nat. Immunol..

[23] Benitez-Ribas D., Adema G.J., Winkels G., Klasen I.S., Punt C.J., Figdor C.G., de Vries I.J. (2006). Plasmacytoid dendritic cells of melanoma patients present exogenous proteins to CD4+ T cells after Fc gamma RII-mediated uptake. J. Exp. Med..

[24] Dzionek A., Fuchs A., Schmidt P., Cremer S., Zysk M., Miltenyi S., Buck D.W., Schmitz J. (2000). BDCA-2, BDCA-3, and BDCA-4: Three markers for distinct subsets of dendritic cells in human peripheral blood. J. Immunol..

[25] Segura E., Valladeau-Guilemond J., Donnadieu M.H., Sastre-Garau X., Soumelis V., Amigorena S. (2012). Characterization of resident and migratory dendritic cells in human lymph nodes. J. Exp. Med..

[26] Merad M., Sathe P., Helft J., Miller J., Mortha A. (2013). The dendritic cell lineage: Ontogeny and function of dendritic cells and their subsets in the steady state and the inflamed setting. Annu. Rev. Immunol..

[27] Macri C., Dumont C., Johnston A.P., Mintern J.D. (2016). Targeting dendritic cells: A promising strategy to improve vaccine effectiveness. Clin. Transl. Immunol..

[28] Caminschi I., Proietto A.I., Ahmet F., Kitsoulis S., Shin Teh J., Lo J.C., Rizzitelli A., Wu L., Vremec D., van Dommelen S.L. (2008). The dendritic cell subtype-restricted C-type lectin Clec9A is a target for vaccine enhancement. Blood.

[29] Shortman K., Heath W.R. (2010). The CD8+ dendritic cell subset. Immunol. Rev..

[30] den Haan J.M., Lehar S.M., Bevan M.J. (2000). CD8(+) but not CD8(-) dendritic cells cross-prime cytotoxic T cells in vivo. J. Exp. Med..

[31] Edwards A.D., Diebold S.S., Slack E.M., Tomizawa H., Hemmi H., Kaisho T., Akira S., Reis e Sousa C. (2003). Toll-like receptor expression in murine DC subsets: Lack of TLR7 expression by CD8 alpha+ DC correlates with unresponsiveness to imidazoquinolines. Eur. J. Immunol..

[32] Kawai T., Akira S. (2007). Signaling to NF-kappaB by Toll-like receptors. Trends Mol. Med..

[33] Heil F., Hemmi H., Hochrein H., Ampenberger F., Kirschning C., Akira S., Lipford G., Wagner H., Bauer S. (2004). Species-specific recognition of single-stranded RNA via toll-like receptor 7 and 8. Science.

[34] Zhang Y., El-Far M., Dupuy F.P., Abdel-Hakeem M.S., He Z., Procopio F.A., Shi Y., Haddad E.K., Ancuta P., Sekaly R.P. (2016). HCV RNA activates APCs via TLR7/TLR8 while virus selectively stimulates macrophages without inducing antiviral responses. Sci. Rep..

[35] Kaplan D.H. (2010). In vivo function of Langerhans cells and dermal dendritic cells. Trends Immunol..

[36] van Kooyk Y., Rabinovich G.A. (2008). Protein-glycan interactions in the control of innate and adaptive immune responses. Nat. Immunol..

[37] Sancho D., Reis e Sousa C. (2013). Sensing of cell death by myeloid C-type lectin receptors. Curr. Opin. Immunol..

[38] Dambuza I.M., Brown G.D. (2015). C-type lectins in immunity: Recent developments. Curr. Opin. Immunol..

[39] Robinson M.J., Sancho D., Slack E.C., LeibundGut-Landmann S., Reis e Sousa C. (2006). Myeloid C-type lectins in innate immunity. Nat. Immunol..

[40] Szolnoky G., Bata-Csorgo Z., Kenderessy A.S., Kiss M., Pivarcsi A., Novak Z., Nagy Newman K., Michel G., Ruzicka T., Marodi L. (2001). A mannose-binding receptor is expressed on human keratinocytes and mediates killing of Candida albicans. J. Investig. Dermatol..

[41] Martinez-Pomares L. (2012). The mannose receptor. J. Leukoc. Biol..

[42] Schlesinger P.H., Doebber T.W., Mandell B.F., White R., DeSchryver C., Rodman J.S., Miller M.J., Stahl P. (1978). Plasma clearance of glycoproteins with terminal mannose and *N*-acetylglucosamine by liver non-parenchymal cells. Studies with β-glucuronidase, *N*-acetyl-β-d-glucosaminidase, ribonuclease B and agalacto-orosomucoid. Biochem. J..

[43] Stahl P.D., Ezekowitz R.A. (1998). The mannose receptor is a pattern recognition receptor involved in host defense. Curr. Opin. Immunol..

[44] Apostolopoulos V., Pietersz G.A., Loveland B.E., Sandrin M.S., McKenzie I.F. (1995). Oxidative/reductive conjugation of mannan to antigen selects for T1 or T2 immune responses. Proc. Natl. Acad. Sci. USA.

[45] Vassilaros S., Tsibanis A., Tsikkinis A., Pietersz G.A., McKenzie I.F., Apostolopoulos V. (2013). Up to 15-year clinical follow-up of a pilot Phase III immunotherapy study in stage II breast cancer patients using oxidized mannan-MUC1. Immunotherapy.

[46] He L.Z., Weidlick J., Sisson C., Marsh H.C., Keler T. (2015). Toll-like receptor agonists shape the immune responses to a mannose receptor-targeted cancer vaccine. Cell. Mol. Immunol..

[47] Shrimpton R.E., Butler M., Morel A.S., Eren E., Hue S.S., Ritter M.A. (2009). CD205 (DEC-205): A recognition receptor for apoptotic and necrotic self. Mol. Immunol..

[48] Kato M., McDonald K.J., Khan S., Ross I.L., Vuckovic S., Chen K., Munster D., MacDonald K.P., Hart D.N. (2006). Expression of human DEC-205 (CD205) multilectin receptor on leukocytes. Int. Immunol..

[49] Jiang W., Swiggard W.J., Heufler C., Peng M., Mirza A., Steinman R.M., Nussenzweig M.C. (1995). The receptor DEC-205 expressed by dendritic cells and thymic epithelial cells is involved in antigen processing. Nature.

[50] Dudziak D., Kamphorst A.O., Heidkamp G.F., Buchholz V.R., Trumpfheller C., Yamazaki S., Cheong C., Liu K., Lee H.W., Park C.G. (2007). Differential antigen processing by dendritic cell subsets in vivo. Science.

[51] Wang B., Zaidi N., He L.Z., Zhang L., Kuroiwa J.M., Keler T., Steinman R.M. (2012). Targeting of the non-mutated tumor antigen HER2/neu to mature dendritic cells induces an integrated immune response that protects against breast cancer in mice. Breast Cancer Res..

[52] Idoyaga J., Lubkin A., Fiorese C., Lahoud M.H., Caminschi I., Huang Y., Rodriguez A., Clausen B.E., Park C.G., Trumpfheller C. (2011). Comparable T helper 1 (Th1) and CD8 T-cell immunity by targeting HIV gag p24 to CD8 dendritic cells within antibodies to Langerin, DEC205, and Clec9A. Proc. Natl. Acad. Sci. USA.

[53] Charalambous A., Oks M., Nchinda G., Yamazaki S., Steinman R.M. (2006). Dendritic cell targeting of survivin protein in a xenogeneic form elicits strong CD4+ T cell immunity to mouse survivin. J. Immunol..

[54] Bonifaz L.C., Bonnyay D.P., Charalambous A., Darguste D.I., Fujii S., Soares H., Brimnes M.K., Moltedo B., Moran T.M., Steinman R.M. (2004). In vivo targeting of antigens to maturing dendritic cells via the DEC-205 receptor improves T cell vaccination. J. Exp. Med..

[55] Hawiger D., Inaba K., Dorsett Y., Guo M., Mahnke K., Rivera M., Ravetch J.V., Steinman R.M., Nussenzweig M.C. (2001). Dendritic cells induce peripheral T cell unresponsiveness under steady state conditions in vivo. J. Exp. Med..

[56] Cheong C., Choi J.H., Vitale L., He L.Z., Trumpfheller C., Bozzacco L., Do Y., Nchinda G., Park S.H., Dandamudi D.B. (2010). Improved cellular and humoral immune responses in vivo following targeting of HIV Gag to dendritic cells within human anti-human DEC205 monoclonal antibody. Blood.

[57] Lahoud M.H., Ahmet F., Zhang J.G., Meuter S., Policheni A.N., Kitsoulis S., Lee C.N., O’Keeffe M., Sullivan L.C., Brooks A.G. (2012). DEC-205 is a cell surface receptor for CpG oligonucleotides. Proc. Natl. Acad. Sci. USA.

[58] Park C.G., Rodriguez A., Ueta H., Lee H., Pack M., Matsuno K., Steinman R.M. (2012). Generation of anti-human DEC205/CD205 monoclonal antibodies that recognize epitopes conserved in different mammals. J. Immunol. Methods.

[59] Chen B.Y., Zhou G., Li Q.L., Lu J.S., Shi D.Y., Pang X.B., Zhou X.W., Yu Y.Z., Huang P.T. (2017). Enhanced effects of DNA vaccine against botulinum neurotoxin serotype A by targeting antigen to dendritic cells. Immunol. Lett..

[60] Beckman R.A., Weiner L.M., Davis H.M. (2007). Antibody constructs in cancer therapy: Protein engineering strategies to improve exposure in solid tumors. Cancer.

[61] Birkholz K., Schwenkert M., Kellner C., Gross S., Fey G., Schuler-Thurner B., Schuler G., Schaft N., Dorrie J. (2010). Targeting of DEC-205 on human dendritic cells results in efficient MHC class II-restricted antigen presentation. Blood.

[62] Lakhrif Z., Moreau A., Herault B., Di-Tommaso A., Juste M., Moire N., Dimier-Poisson I., Mevelec M.N., Aubrey N. (2018). Targeted delivery of *Toxoplasma gondii* antigens to dendritic cells promote immunogenicity and protective efficiency against *Toxoplasmosis*. Front. Immunol..

[63] Ngu L.N., Nji N.N., Ambada G.E., Sagnia B., Sake C.N., Tchadji J.C., Njambe Priso G.D., Lissom A., Tchouangueu T.F., Manga Tebit D. (2017). In vivo targeting of protein antigens to dendritic cells using anti-DEC-205 single chain antibody improves HIV Gag specific CD4(+) T cell responses protecting from airway challenge with recombinant vaccinia-gag virus. Immun. Inflamm. Dis..

[64] Kim J.W., Kane J.R., Panek W.K., Young J.S., Rashidi A., Yu D., Kanojia D., Hasan T., Miska J., Gomez-Lim M.A. (2018). A dendritic cell-targeted adenoviral vector facilitates adaptive immune response against human glioma antigen (CMV-IE) and prolongs survival in a human glioma tumor model. Neurotherapeutics.

[65] Antonio-Herrera L., Badillo-Godinez O., Medina-Contreras O., Tepale-Segura A., Garcia-Lozano A., Gutierrez-Xicotencatl L., Soldevila G., Esquivel-Guadarrama F.R., Idoyaga J., Bonifaz L.C. (2018). The nontoxic cholera B subunit Is a potent adjuvant for intradermal DC-targeted vaccination. Front. Immunol..

[66] Reid D.M., Gow N.A., Brown G.D. (2009). Pattern recognition: Recent insights from Dectin-1. Curr. Opin. Immunol..

[67] Brown G.D. (2006). Dectin-1: A signalling non-TLR pattern-recognition receptor. Nat. Rev. Immunol..

[68] Brown G.D., Gordon S. (2001). Immune recognition. A new receptor for β-glucans. Nature.

[69] Monteiro J.T., Lepenies B. (2017). Myeloid C-type lectin receptors in viral recognition and antiviral immunity. Viruses.

[70] Dennehy K.M., Brown G.D. (2007). The role of the β-glucan receptor Dectin-1 in control of fungal infection. J. Leukoc. Biol..

[71] Rivera A., Hohl T.M., Collins N., Leiner I., Gallegos A., Saijo S., Coward J.W., Iwakura Y., Pamer E.G. (2011). Dectin-1 diversifies Aspergillus fumigatus-specific T cell responses by inhibiting T helper type 1 CD4 T cell differentiation. J. Exp. Med..

[72] Hernandez-Santos N., Gaffen S.L. (2012). Th17 cells in immunity to Candida albicans. Cell Host Microbe.

[73] Leibundgut-Landmann S., Osorio F., Brown G.D., Reis e Sousa C. (2008). Stimulation of dendritic cells via the dectin-1/Syk pathway allows priming of cytotoxic T-cell responses. Blood.

[74] Graham L.M., Brown G.D. (2009). The Dectin-2 family of C-type lectins in immunity and homeostasis. Cytokine.

[75] Kimura Y., Inoue A., Hangai S., Saijo S., Negishi H., Nishio J., Yamasaki S., Iwakura Y., Yanai H., Taniguchi T. (2016). The innate immune receptor Dectin-2 mediates the phagocytosis of cancer cells by Kupffer cells for the suppression of liver metastasis. Proc. Natl. Acad. Sci. USA.

[76] Chiffoleau E. (2018). C-type lectin-like receptors as emerging orchestrators of sterile inflammation represent potential therapeutic targets. Front. Immunol..

[77] Kerrigan A.M., Brown G.D. (2009). C-type lectins and phagocytosis. Immunobiology.

[78] Appelmelk B.J., van Die I., van Vliet S.J., Vandenbroucke-Grauls C.M., Geijtenbeek T.B., van Kooyk Y. (2003). Cutting edge: Carbohydrate profiling identifies new pathogens that interact with dendritic cell-specific ICAM-3-grabbing nonintegrin on dendritic cells. J. Immunol..

[79] Engering A., Geijtenbeek T.B., van Vliet S.J., Wijers M., van Liempt E., Demaurex N., Lanzavecchia A., Fransen J., Figdor C.G., Piguet V. (2002). The dendritic cell-specific adhesion receptor DC-SIGN internalizes antigen for presentation to T cells. J. Immunol..

[80] Geijtenbeek T.B., Kwon D.S., Torensma R., van Vliet S.J., van Duijnhoven G.C., Middel J., Cornelissen I.L., Nottet H.S., KewalRamani V.N., Littman D.R. (2000). DC-SIGN, a dendritic cell-specific HIV-1-binding protein that enhances trans-infection of T cells. Cell.

[81] van Dinther D., Stolk D.A., van de Ven R., van Kooyk Y., de Gruijl T.D., den Haan J.M.M. (2017). Targeting C-type lectin receptors: A high-carbohydrate diet for dendritic cells to improve cancer vaccines. J. Leukoc. Biol..

[82] Cruz L.J., Tacken P.J., Fokkink R., Joosten B., Stuart M.C., Albericio F., Torensma R., Figdor C.G. (2010). Targeted PLGA nano- but not microparticles specifically deliver antigen to human dendritic cells via DC-SIGN in vitro. J. Control. Release.

[83] Hesse C., Ginter W., Forg T., Mayer C.T., Baru A.M., Arnold-Schrauf C., Unger W.W., Kalay H., van Kooyk Y., Berod L. (2013). In vivo targeting of human DC-SIGN drastically enhances CD8(+) T-cell-mediated protective immunity. Eur. J. Immunol..

[84] Kretz-Rommel A., Qin F., Dakappagari N., Torensma R., Faas S., Wu D., Bowdish K.S. (2007). In vivo targeting of antigens to human dendritic cells through DC-SIGN elicits stimulatory immune responses and inhibits tumor growth in grafted mouse models. J. Immunother..

[85] Tacken P.J., Joosten B., Reddy A., Wu D., Eek A., Laverman P., Kretz-Rommel A., Adema G.J., Torensma R., Figdor C.G. (2008). No advantage of cell-penetrating peptides over receptor-specific antibodies in targeting antigen to human dendritic cells for cross-presentation. J. Immunol..

[86] Cruz F.M., Colbert J.D., Merino E., Kriegsman B.A., Rock K.L. (2017). The biology and underlying mechanisms of cross-presentation of exogenous antigens on MHC-I molecules. Annu. Rev. Immunol..

[87] Ahrens S., Zelenay S., Sancho D., Hanc P., Kjaer S., Feest C., Fletcher G., Durkin C., Postigo A., Skehel M. (2012). F-actin is an evolutionarily conserved damage-associated molecular pattern recognized by DNGR-1, a receptor for dead cells. Immunity.

[88] Sancho D., Mourao-Sa D., Joffre O.P., Schulz O., Rogers N.C., Pennington D.J., Carlyle J.R., Reis e Sousa C. (2008). Tumor therapy in mice via antigen targeting to a novel, DC-restricted C-type lectin. J. Clin. Investig..

[89] Picco G., Beatson R., Taylor-Papadimitriou J., Burchell J.M. (2014). Targeting DNGR-1 (CLEC9A) with antibody/MUC1 peptide conjugates as a vaccine for carcinomas. Eur. J. Immunol..

[90] Joffre O.P., Sancho D., Zelenay S., Keller A.M., Reis e Sousa C. (2010). Efficient and versatile manipulation of the peripheral CD4+ T-cell compartment by antigen targeting to DNGR-1/CLEC9A. Eur. J. Immunol..

[91] Li J., Ahmet F., Sullivan L.C., Brooks A.G., Kent S.J., De Rose R., Salazar A.M., Reis e Sousa C., Shortman K., Lahoud M.H. (2015). Antibodies targeting Clec9A promote strong humoral immunity without adjuvant in mice and non-human primates. Eur. J. Immunol..

[92] Schulz O., Hanc P., Bottcher J.P., Hoogeboom R., Diebold S.S., Tolar P., Reis E.S.C. (2018). Myosin II synergizes with F-Actin to promote DNGR-1-dependent cross-presentation of dead cell-associated antigens. Cell Rep..

[93] Nimmerjahn F., Ravetch J.V. (2008). Fcgamma receptors as regulators of immune responses. Nat. Rev. Immunol..

[94] Sanchez-Mejorada G., Rosales C. (1998). Signal transduction by immunoglobulin Fc receptors. J. Leukoc. Biol..

[95] Pincetic A., Bournazos S., DiLillo D.J., Maamary J., Wang T.T., Dahan R., Fiebiger B.M., Ravetch J.V. (2014). Type I and type II Fc receptors regulate innate and adaptive immunity. Nat. Immunol..

[96] Bournazos S., Ravetch J.V. (2017). Fcgamma receptor function and the design of vaccination strategies. Immunity.

[97] Swanson J.A., Hoppe A.D. (2004). The coordination of signaling during Fc receptor-mediated phagocytosis. J. Leukoc. Biol..

[98] Roopenian D.C., Akilesh S. (2007). FcRn: The neonatal Fc receptor comes of age. Nat. Rev. Immunol..

[99] Mallery D.L., McEwan W.A., Bidgood S.R., Towers G.J., Johnson C.M., James L.C. (2010). Antibodies mediate intracellular immunity through tripartite motif-containing 21 (TRIM21). Proc. Natl. Acad. Sci. USA.

[100] Guilliams M., Bruhns P., Saeys Y., Hammad H., Lambrecht B.N. (2014). The function of Fcgamma receptors in dendritic cells and macrophages. Nat. Rev. Immunol..

[101] Pham G.H., Iglesias B.V., Gosselin E.J. (2014). Fc receptor-targeting of immunogen as a strategy for enhanced antigen loading, vaccination, and protection using intranasally administered antigen-pulsed dendritic cells. Vaccine.

[102] Bitsaktsis C., Babadjanova Z., Gosselin E.J. (2015). In vivo mechanisms involved in enhanced protection utilizing an Fc receptor-targeted mucosal vaccine platform in a bacterial vaccine and challenge model. Infect. Immun..

[103] de Jong J.M., Schuurhuis D.H., Ioan-Facsinay A., van der Voort E.I., Huizinga T.W., Ossendorp F., Toes R.E., Verbeek J.S. (2006). Murine Fc receptors for IgG are redundant in facilitating presentation of immune complex derived antigen to CD8+ T cells in vivo. Mol. Immunol..

[104] Hossain M.K., Wall K.A. (2016). Immunological evaluation of recent MUC1 glycopeptide cancer vaccines. Vaccines.

[105] Lakshminarayanan V., Supekar N.T., Wei J., McCurry D.B., Dueck A.C., Kosiorek H.E., Trivedi P.P., Bradley J.M., Madsen C.S., Pathangey L.B. (2016). MUC1 vaccines, comprised of glycosylated or non-glycosylated peptides or tumor-derived MUC1, can circumvent immunoediting to control tumor growth in MUC1 transgenic mice. PLoS ONE.

[106] Gong J., Apostolopoulos V., Chen D., Chen H., Koido S., Gendler S.J., McKenzie I.F., Kufe D. (2000). Selection and characterization of MUC1-specific CD8+ T cells from MUC1 transgenic mice immunized with dendritic-carcinoma fusion cells. Immunology.

[107] Karmakar P., Lee K., Sarkar S., Wall K.A., Sucheck S.J. (2016). Synthesis of a Liposomal MUC1 glycopeptide-based immunotherapeutic and evaluation of the effect of l-Rhamnose targeting on cellular immune responses. Bioconjug. Chem..

[108] Hossain M.K., Vartak A., Karmakar P., Sucheck S.J., Wall K.A. (2018). Augmenting vaccine immunogenicity through the use of natural human anti-rhamnose antibodies. ACS Chem. Biol..

[109] Ngu L.N., Nji N.N., Ambada G., Ngoh A.A., Njambe Priso G.D., Tchadji J.C., Lissom A., Magagoum S.H., Sake C.N., Tchouangueu T.F. (2018). Dendritic cell targeted HIV-1 gag protein vaccine provides help to a recombinant Newcastle disease virus vectored vaccine including mobilization of protective CD8(+) T cells. Immun. Inflamm. Dis..

[110] Hua Y., Jiao Y.Y., Ma Y., Peng X.L., Fu Y.H., Zhang X.J., Zheng Y.B., Zheng Y.P., Hong T., He J.S. (2017). Enhanced humoral and CD8+ T cell immunity in mice vaccinated by DNA vaccine against human respiratory syncytial virus through targeting the encoded F protein to dendritic cells. Int. Immunopharmacol..

[111] Glaffig M., Stergiou N., Hartmann S., Schmitt E., Kunz H. (2018). A synthetic MUC1 anticancer vaccine containing mannose ligands for targeting macrophages and dendritic cells. ChemMedChem.

[112] Silva J.M., Zupancic E., Vandermeulen G., Oliveira V.G., Salgado A., Videira M., Gaspar M., Graca L., Preat V., Florindo H.F. (2015). In vivo delivery of peptides and Toll-like receptor ligands by mannose-functionalized polymeric nanoparticles induces prophylactic and therapeutic anti-tumor immune responses in a melanoma model. J. Control. Release.

[113] Dzharullaeva A.S., Tukhvatulin A.I., Erokhova A.S., Bandelyuk A.S., Polyakov N.B., Solovyev A.I., Nikitenko N.A., Shcheblyakov D.V., Naroditsky B.S., Logunov D.Y. (2018). Stimulation of Dectin-1 and Dectin-2 during Parenteral Immunization, but Not Mincle, Induces Secretory IgA in Intestinal Mucosa. J. Immunol. Res..

[114] Donadei A., Gallorini S., Berti F., O’Hagan D.T., Adamo R., Baudner B.C. (2015). Rational design of adjuvant for skin delivery: Conjugation of synthetic β-glucan dectin-1 agonist to protein antigen. Mol. Pharm..

[115] Velasquez L.N., Stuve P., Gentilini M.V., Swallow M., Bartel J., Lycke N.Y., Barkan D., Martina M., Lujan H.D., Kalay H. (2018). Targeting *Mycobacterium tuberculosis* antigens to dendritic cells via the DC-specific-ICAM3-Grabbing-nonintegrin receptor induces strong T-Helper 1 immune responses. Front. Immunol..

[116] Le Moignic A., Malard V., Benvegnu T., Lemiegre L., Berchel M., Jaffres P.A., Baillou C., Delost M., Macedo R., Rochefort J. (2018). Preclinical evaluation of mRNA trimannosylated lipopolyplexes as therapeutic cancer vaccines targeting dendritic cells. J. Control. Release.

[117] Caminschi I., Vremec D., Ahmet F., Lahoud M.H., Villadangos J.A., Murphy K.M., Heath W.R., Shortman K. (2012). Antibody responses initiated by Clec9A-bearing dendritic cells in normal and Batf3(-/-) mice. Mol. Immunol..

[118] Gall V.A., Philips A.V., Qiao N., Clise-Dwyer K., Perakis A.A., Zhang M., Clifton G.T., Sukhumalchandra P., Ma Q., Reddy S.M. (2017). Trastuzumab increases HER2 uptake and cross-presentation by dendritic cells. Cancer Res..

[119] Abdel-Motal U.M., Wang S., Awad A., Lu S., Wigglesworth K., Galili U. (2010). Increased immunogenicity of HIV-1 p24 and gp120 following immunization with gp120/p24 fusion protein vaccine expressing alpha-gal epitopes. Vaccine.

[120] Abdel-Motal U.M., Guay H.M., Wigglesworth K., Welsh R.M., Galili U. (2007). Immunogenicity of influenza virus vaccine is increased by anti-gal-mediated targeting to antigen-presenting cells. J. Virol..

